# Supplementary material for: Single-cell analysis reveals prognostic fibroblast subpopulations linked to molecular and immunological subtypes of lung cancer
Source: Nat Commun. 2023 Jan 31;14:387. doi: 10.1038/s41467-023-35832-6 (PMC9889778; doi:10.1038/s41467-023-35832-6)
Supplement: Supplementary file 1 — Supplementary Information [file 41467_2023_35832_MOESM1_ESM.pdf]

Single-cell analysis reveals prognostic fibroblast subpopulations linked to molecular and immunological subtypes of lung cancer (Supplementary Information)

Christopher J. Hanley[1,2#], Sara Waise[1*], Matthew J. Ellis[1*], Maria A. Lopez[3], Wai Y. Pun[3], Julian Taylor[3], Rachel Parker[1], Lucy M. Kimbley[1], Serena J. Chee[1,4], Emily C. Shaw[3], Jonathan West[1,5], Aiman Alzetani[6], Edwin Woo[6], Christian H. Ottensmeier[1,2,4], Matthew J.J. Rose-Zerilli[1,5], Gareth J. Thomas[1,2,3#]

[1] School of Cancer Sciences, University of Southampton, Southampton, UK, SO16 6YD.

[2] Cancer Research UK and NIHR Southampton Experimental Cancer Medicine Centre, Southampton, UK, SO16 6YD.

[3] Department of Histopathology, University Hospital Southampton NHS Foundation Trust, Southampton, UK, SO16 6YD.

[4] Institute of Systems, Molecular and Integrative Biology (ISMIB) and Liverpool Experimental Cancer Medicines Centre, University of Liverpool, Liverpool UK, L69 7BE.

[5] Institute for Life Sciences, University of Southampton, Southampton, UK, SO17 1BJ.

[6] Department of Thoracic surgery, University Hospital Southampton NHS Foundation Trust, UK, SO16 6YD.

[#]Corresponding authors; *Contributed equally

Corresponding authors:

Christopher Hanley (C.J.Hanley@soton.ac.uk) and Gareth Thomas (G.Thomas@soton.ac.uk)

Supplementary Figures

Supplementary Figure 1: Fibroblast identification through single-cell RNA sequencing analysis of whole-tissue homogenates derived from human NSCLC tumour samples.

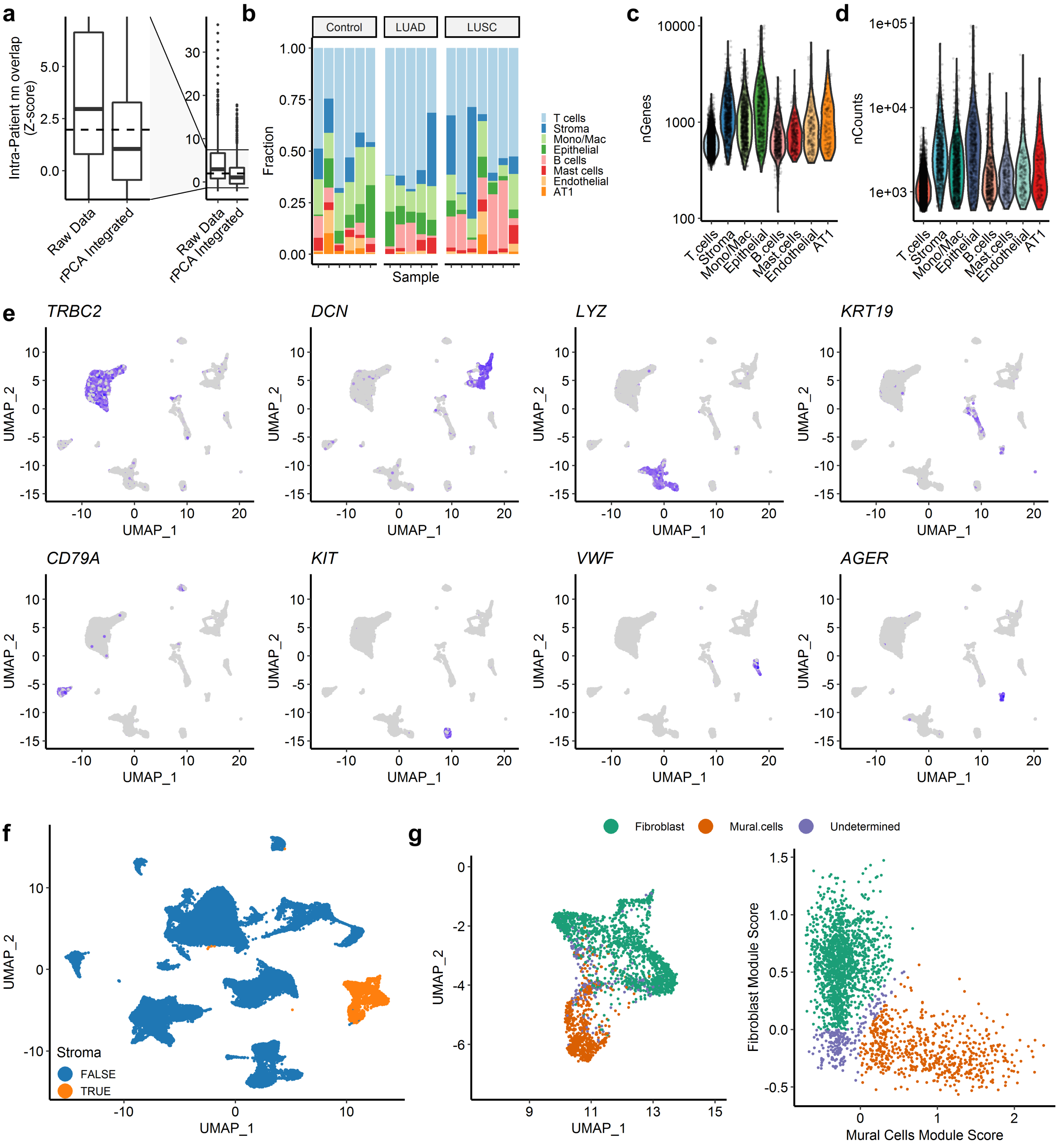

a) Boxplots showing the results of batch correction testing after reciprocal PCA (rPCA) integration (n = 8158 single cell transcriptomes). Intra-sample nearest neighbour (nn) overlap z-scores define whether the cells from individual samples are grouped together with greater frequency than randomly sampled cells, z-scores greater than 1.96 (equivalent to p=0.05) indicate cells that significantly overlap with other cells from the same dataset and this threshold is shown by the dotted horizontal line.

b) Barplot showing the relative abundance of different cell types across all samples in the TLDS scRNA-seq dataset.
c) Violin plot showing the number of genes measure per cell, grouped by cell type.
d) Violin plot showing the number of unique molecular identifiers (counts) measured per cell, grouped by cell type.
e) Feature plots showing the expression of canonical cell type markers: *TRBC2* (T cells); *DCN* (Fibroblasts); *LYZ* (Myeloid cells); *KRT19* (Epithelial cells); *CD79A* (B cells); *KIT* (Mast cells); *VWF* (Endothelial cells); *AGER* (AT1 pneumocytes).
f) 2D visualisation (UMAP dimensionality reduction) of Qian *et al.'s* scRNA-seq data [1], highlighting the stromal cell cluster.
g) UMAP and scatter plot showing the demarcation of mural cells and fibroblasts within the Qian dataset's stromal cell cluster, using consensus fibroblast and mural cell gene signature scores.

All boxplots are displayed using the Tukey method (centre line, median; box limits, upper and lower quartiles; whiskers, last point within a 1.5x interquartile range).

Supplementary Figure 2: Integration of seven scRNA-seq datasets identifies three major fibroblast subpopulations in human NSCLC and control tissues.

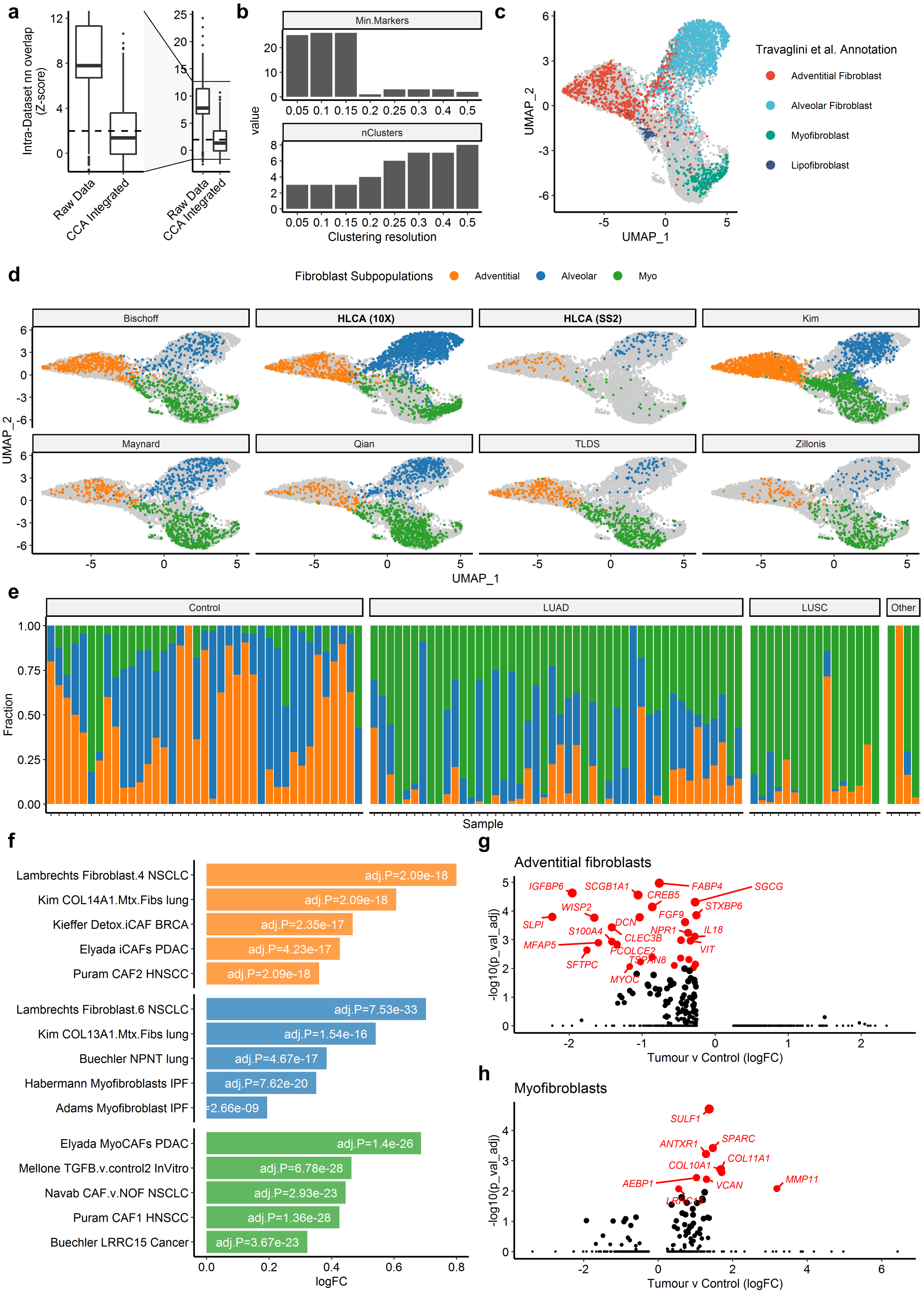

a) Boxplots showing the intra-dataset nearest neighbour (nn) overlap z-score before and after canonical correlation analysis (CCA) mediated integration (n = 9673 fibroblast transcriptomes). Intra-dataset nearest neighbour (nn) overlap z-scores define whether the cells from individual datasets are grouped together with greater frequency than randomly sampled cells, z-scores greater than 1.96 (equivalent to p=0.05) indicate cells that significantly overlap with other cells from the same dataset and this threshold is shown by the dotted horizontal line.
b) Bar plots showing the number of clusters and minimum number of sample-level markers per cluster for different clustering resolutions, resolution 0.15 was selected for fibroblast subpopulation identification.
c) UMAP plot showing the distribution of fibroblasts from the HLCA dataset[2] and their original annotations.
d) UMAP plots showing the distribution of fibroblasts from each of datasets used.
e) Bar plot showing the relative abundance of fibroblast subpopulations grouped by tissue subtype.
f) Bar plot showing the most significantly upregulated fibroblast gene signatures for each subpopulation, calculated through GSVA. Complete results from this analysis are provided in Supplementary Data 4.
g) Volcano plot showing sample level differential expression analysis between adventitial fibroblasts isolated from tumour samples or control samples. Genes differentially expressed (adj.*P*<0.01, shown in red and labelled, n = 36 [Control], 42 [Tumour]). Complete results from this analysis are provided in Supplementary Data 5.
h) Volcano plot showing sample level differential expression analysis between myofibroblasts isolated from tumour samples or control samples. Genes differentially expressed (adj.*P*<0.01, shown in red and labelled, n = 28 [Control], 64 [Tumour]). Complete results from this analysis are provided in Supplementary Data 5.

All boxplots are displayed using the Tukey method (centre line, median; box limits, upper and lower quartiles; whiskers, last point within a 1.5x interquartile range).

Supplementary Figure 3: Multiplexed IHC (mxIHC) and digital cytometry show that fibroblast subpopulations occupy spatially discrete niches and varied NSCLC tissue subtype enrichment.

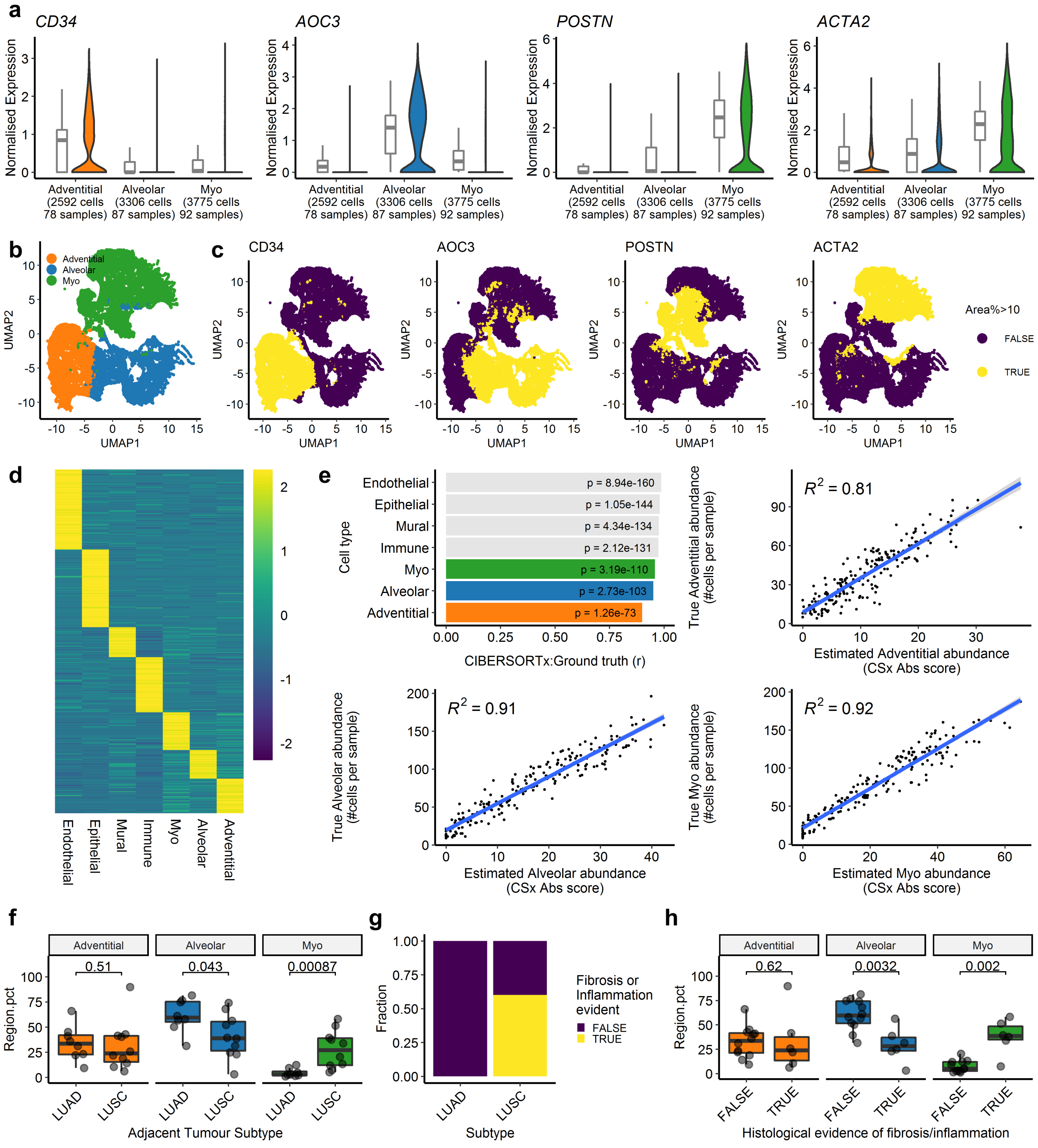

a) Bar and violin plots showing the expression of markers selected to demarcate fibroblast subpopulations by mxIHC. Boxplots represent sample level expression (averaged over single cells) and violin plots show expression at the single cell level.
b) 2D visualisation (UMAP dimensionality reduction) of the fibroblasts identified through mxIHC, highlighting the three subpopulations.
c) Feature plots showing the distribution of fibroblast subpopulation markers over the UMAP dimensionality reduction, as measured by mxIHC.

d) Heatmap showing the signature matrix used for bulk transcriptome deconvolution by CIBERSORTx (CSx), also available in ".txt" format as Supplementary Data 11.
e) CSx accuracy validation using a pseudobulk dataset generated from single cell transcriptomes. Barplot shows the correlation (Pearson's r) between each cell type's CSx estimation and true abundance. Scatter plots show the linear relationship between CSx estimates and true abundance for each fibroblast subpopulation.
f) Boxplot showing the relative abundance of each fibroblast subpopulation in tumour-adjacent normal tissue grouped by the subtype of the associated tumour, measured by mxIHC. Nominal p-values for Wilcoxon signed-ranks test are also shown(n = 8 [LUAD], 10 [LUSC])
g) Barplot showing the relative frequency of inflammation or fibrosis in the tumour-adjacent normal tissue for each tumour subtype. p = 0.00729, chi squared test (n = 8 [LUAD], 10 [LUSC]).
h) Boxplot showing the relative abundance of each fibroblast subpopulation in tumour-adjacent normal tissue (measured by mxIHC) in samples grouped by whether there was histological evidence of inflammation or fibrosis Nominal p-values for Wilcoxon signed-ranks test are also shown (n = 12 [FALSE], 6 [TRUE]).

All statistical tests carried out were two-sided and boxplots are displayed using the Tukey method (centre line, median; box limits, upper and lower quartiles; whiskers, last point within a 1.5x interquartile range). Source data for panels e, f and h are provided in the Source Data file.

Supplementary Figure 4: Trajectory inference identifies consensus gene modules associated with the transdifferentiation from alveolar or adventitial fibroblasts to myofibroblasts.

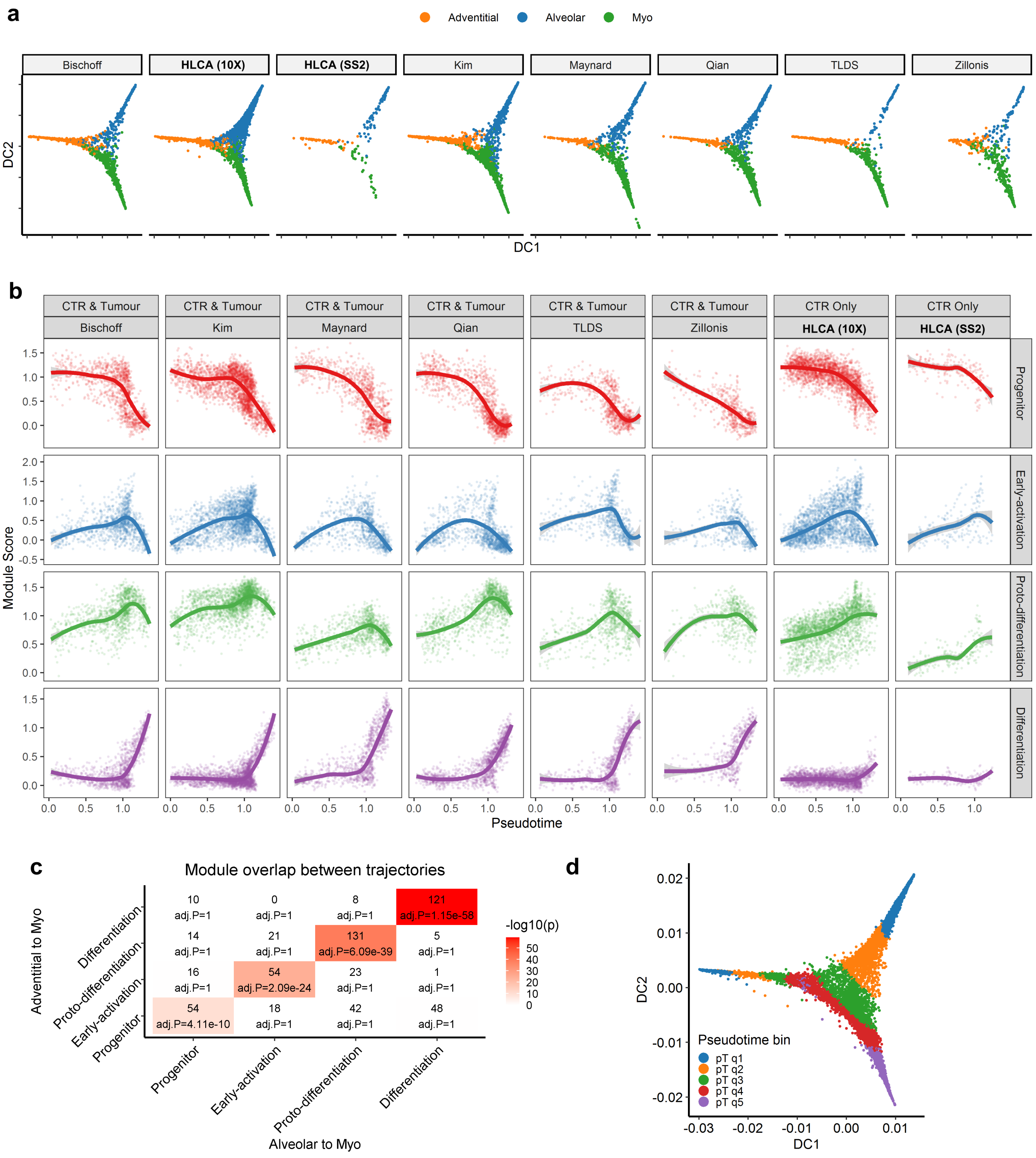

a) 2D visualisation (diffusion map dimensionality reduction) of the integrated fibroblast scRNA-seq dataset split by dataset, highlighting the three subpopulations.

b) Loess plots showing consensus DPT module expression profiles, across each scRNA-seq dataset.

c) Heatmap showing the number of overlapping genes assigned to each DPT module for the alveolar to myo and adventitial to myo trajectory. Benjamini-Hochberg adjusted p-values for Fischer exact tests are also shown.

d) 2D visualisation (diffusion map dimensionality reduction) showing the cells grouped by DPT quintiles.

Supplementary Figure 5: Machine learning based classification of scRNA-seq data and mxIHC shows adventitial and myo-fibroblasts are conserved across cancer types, whereas alveolar fibroblasts are lung specific.

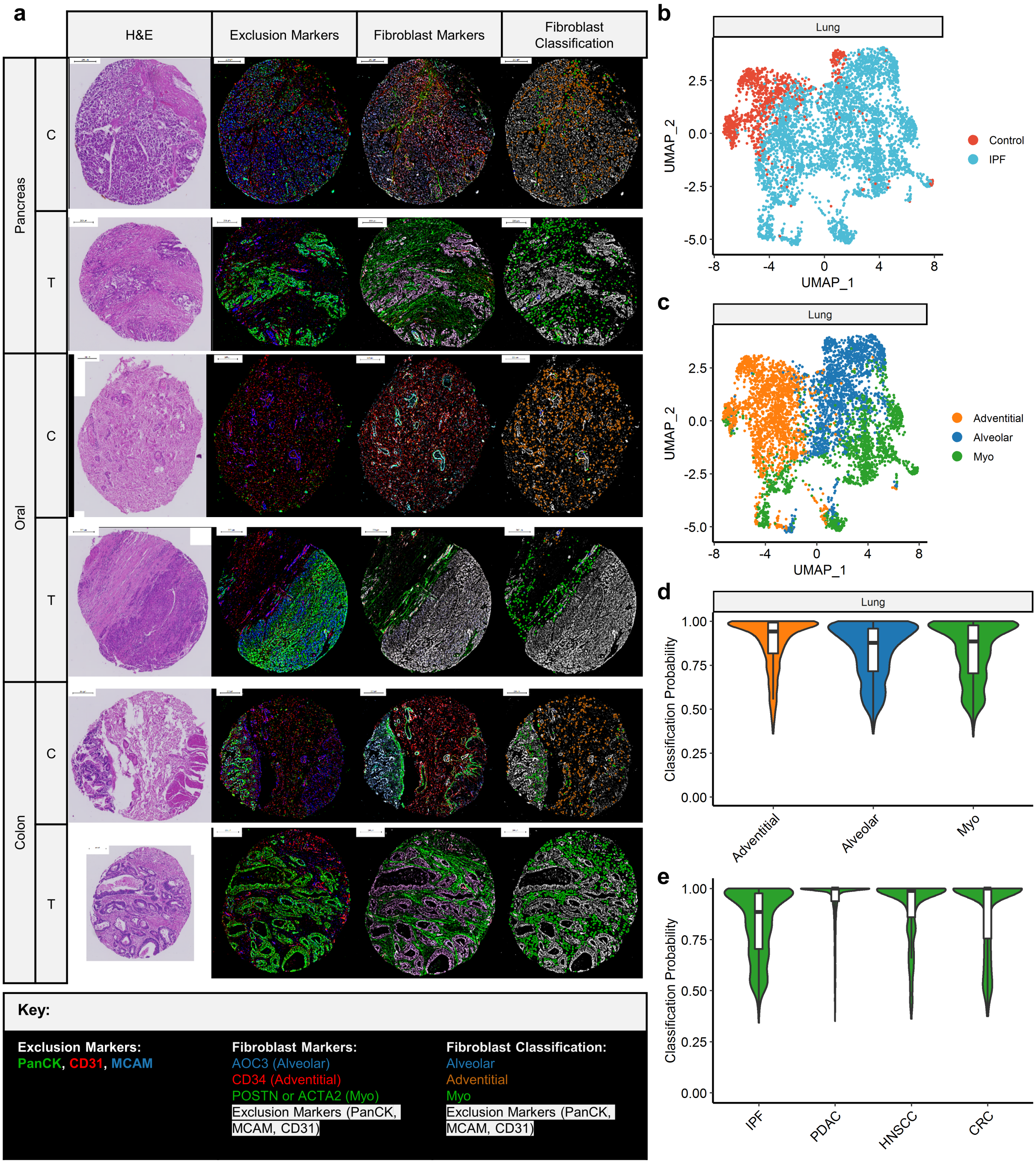

a) Representative micrographs from mxIHC analysis of tissue microarrays (TMAs), constructed from pancreatic, oral and colon cancer tissue blocks (n cores analysed Control/Tumour = 14/15 [Pancreas], 10/9 [Oral], 9/13 [Colon]). The representing each marker or simulated cell classification are provided in the associated key (scale bar represents 100µm).

b) 2D visualisation (UMAP dimensionality reduction) of fibroblasts isolated from idiopathic pulmonary fibrosis (IPF) and control tissues analysed by scRNA-seq, highlighting sample type.
c) As per a, highlighting the fibroblast subpopulation associated with each cell as predicted by a machine learning classifier.
d) Violin plots showing the probability of the machine learning classifier model's predictions (shown in panel c, n = 5066 fibroblast transcriptomes).
e) Violin plots showing the probability of myofibroblast assignments across data from different tissue types (n = 1522 [IPF], 5954 [Pancreas], 413 [Oral], 1885 [Colon] myofibroblast transcriptomes).

All boxplots are displayed using the Tukey method (centre line, median; box limits, upper and lower quartiles; whiskers, last point within a 1.5x interquartile range).

Supplementary Figure 6: CIBERSORTx mediated digital cytometry shows that myofibroblasts and alveolar fibroblasts correlate with overall survival rates in LUAD.

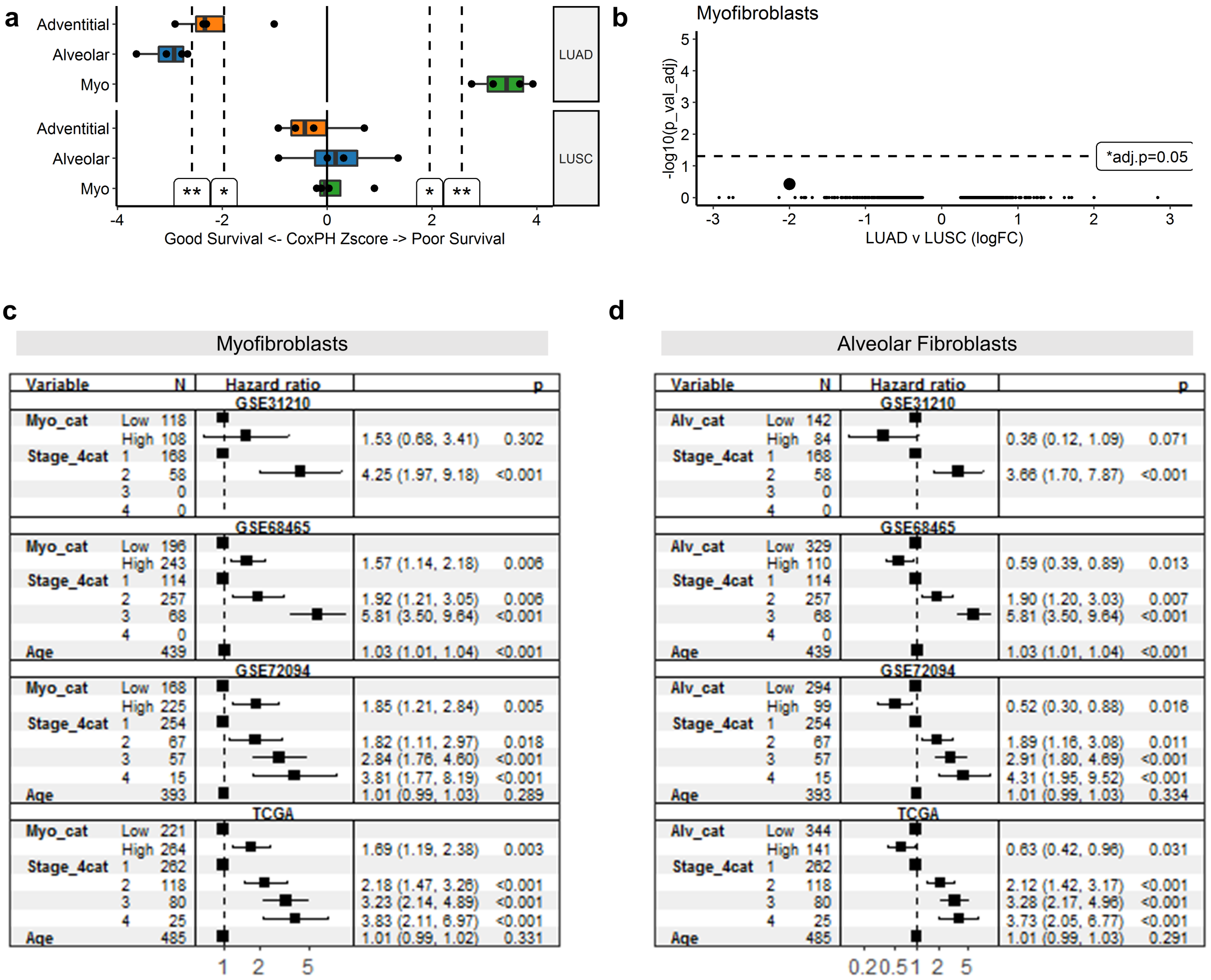

a) Boxplots showing univariate Cox regression analysis of four-year overall survival rates in multiple bulk transcriptomic datasets. Relative fibroblast subpopulation abundance (% of all fibroblasts per sample) was used as a continuous variable for this analysis. Each point represents a different transcriptomic dataset. *p<0.05, **p<0.01 (LUAD datasets n = 503 [TCGA-LUAD[3]], 398 [GSE72094[4]], 442 [GSE68465[5]], 226 [GSE31210[6]]; LUSC datasets n = 492 [TCGA-LUSC[7]], 129 [GSE4573[8]], 234 [GSE157009[9]], 249 [GSE157010[9]]; exact p-values provided in Source Data file).

b) Volcano plot showing sample level differential expression (log fold change and wilcox test Bonferroni adjusted p-values) analysis between myofibroblasts isolated from LUAD samples or LUSC samples (n= 45 [LUAD], 16 [LUSC]).

c) Forest plots showing covariate independent hazard ratios (+/-95% confidence intervals) and adjusted p-values from multivariate Cox regression analysis of four-year overall survival rates across each individual LUAD patient cohort analysed, using myofibroblast

abundance, disease stage and patient age as independent variables (exact p-values provided in Source Data file).

d) Forest plots showing covariate independent hazard ratios (+/-95% confidence intervals) and adjusted p-values each individual LUAD patient cohort analysed[3, 4, 5, 6], using alveolar fibroblast abundance, disease stage and patient age as independent variables (exact p-values provided in Source Data file).

All statistical tests carried out were two-sided and boxplots are displayed using the Tukey method (centre line, median; box limits, upper and lower quartiles; whiskers, last point within a 1.5x interquartile range). Source data for panels a, c and d are provided in the Source Data file.

Supplementary Figure 7: Fibroblast subpopulations are associated with morphological, molecular and immunological features of LUAD tumours.

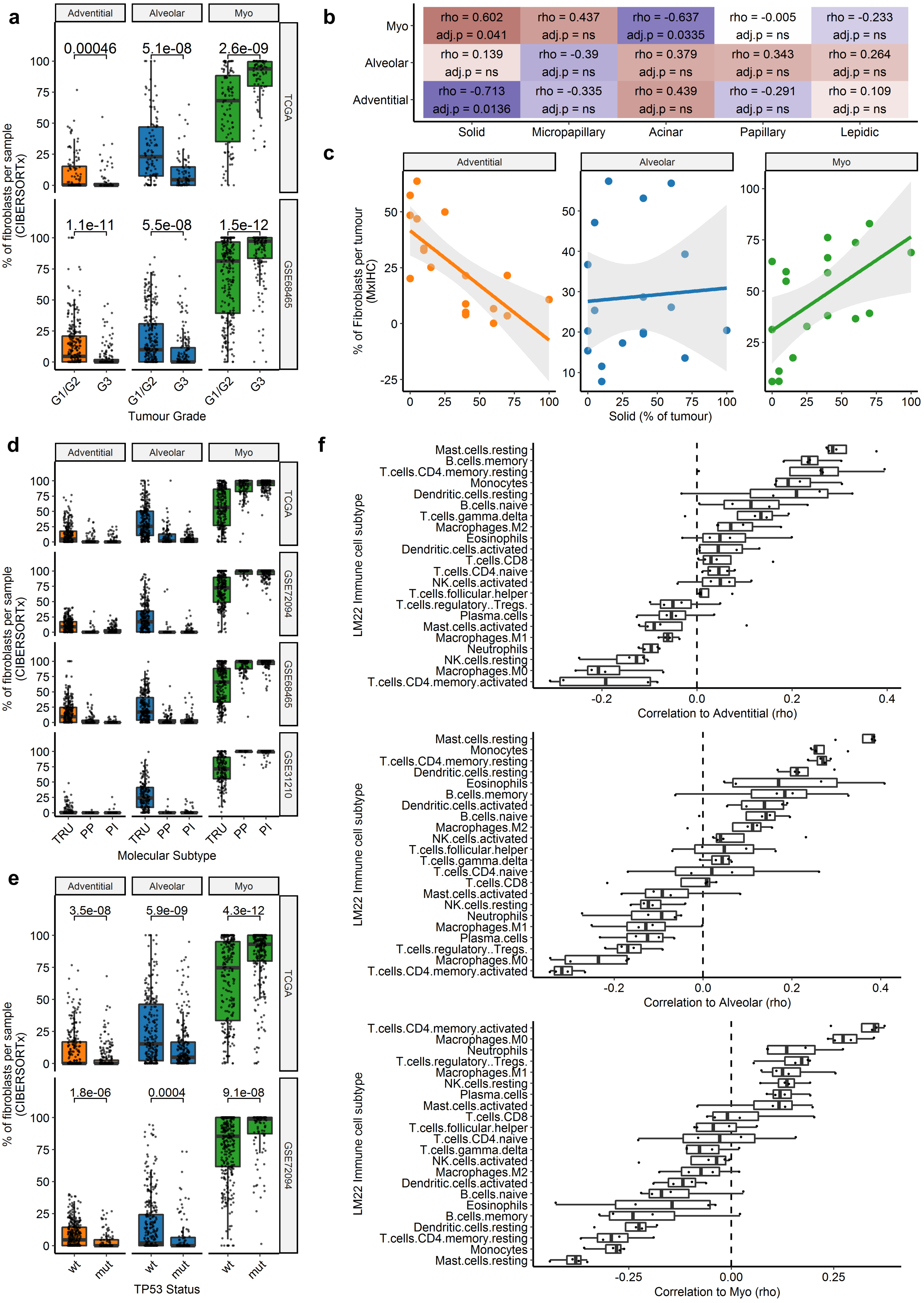

a) Boxplots showing the relative abundance of fibroblast subpopulations in well/moderately differentiated LUAD tumours compared to poorly differentiated, measured by CIBERSORTx digital cytometry. Nominal p-values for Wilcoxon signed-ranks test are also shown (n = 106 [G1/G2-TCGA], 81 [G3-TCGA][3], 269 [G1/G2-GSE68465], 167 [G3-GSE68465][5]).
b) Heatmap showing Spearman's correlation (rho) between fibroblast subpopulations (measured by mxIHC) and LUAD morphological growth patterns (determined by routine clinical pathology). Statistical significance is shown as Benjamini-hochberg adjusted p-values for comparisons where adj.p<0.05 (n = 12).
c) Scatter plots showing the association between each fibroblast subpopulation and the proportion of the tumour comprised of solid morphology growth pattern. Trendline represents a linear regression model with error bands representing 95% confidence intervals.
d) Boxplots showing the relative abundance of fibroblast subpopulations in LUAD tumours grouped by molecular subtype (TRU = Terminal Respiratory Unit, PP = Proximal Proliferative and PI = Proximal Inflammatory), measured by CIBERSORTx digital cytometry (n = 515 [TCGA[3]], 442 [GSE72094[4]], 443 [GSE68465[5]], 226 [GSE31210[6]] LUAD samples).
e) Boxplots showing the relative abundance of fibroblast subpopulations in LUAD tumours grouped by *TP53* mutation status, measured by CIBERSORTx digital cytometry. Nominal p-values for Wilcoxon signed-ranks test are also shown (n = 247 [wt-TCGA], 263 [mut-TCGA][3], 331 [wt-GSE72094], 111 [mut-GSE72094][4]).
f) Scatter plot showing Spearman's correlation between alveolar or myofibroblast abundance and LM22 immune cell subpopulations across LUAD transcriptomic datasets (n = 4 datasets).

All statistical tests carried out were two-sided and boxplots are displayed using the Tukey method (centre line, median; box limits, upper and lower quartiles; whiskers, last point within a 1.5x interquartile range). Source data for panels a, c, d and e are provided in the Source Data file.

Supplementary References

1. Qian J*, et al.* A pan-cancer blueprint of the heterogeneous tumor microenvironment revealed by single-cell profiling. *Cell Res* **30**, 745-762 (2020).

2. Travaglini KJ*, et al.* A molecular cell atlas of the human lung from single-cell RNA sequencing. *Nature* **587**, 619-625 (2020).

3. Cancer Genome Atlas Research N. Comprehensive molecular profiling of lung adenocarcinoma. *Nature* **511**, 543-550 (2014).

4. Schabath MB*, et al.* Differential association of STK11 and TP53 with KRAS mutation-associated gene expression, proliferation and immune surveillance in lung adenocarcinoma. *Oncogene* **35**, 3209-3216 (2016).

5. Director's Challenge Consortium for the Molecular Classification of Lung A*, et al.* Gene expression-based survival prediction in lung adenocarcinoma: a multi-site, blinded validation study. *Nat Med* **14**, 822-827 (2008).

6. Okayama H*, et al.* Identification of genes upregulated in ALK-positive and EGFR/KRAS/ALK-negative lung adenocarcinomas. *Cancer Res* **72**, 100-111 (2012).

7. Cancer Genome Atlas Research N. Comprehensive genomic characterization of squamous cell lung cancers. *Nature* **489**, 519-525 (2012).

8. Raponi M*, et al.* Gene expression signatures for predicting prognosis of squamous cell and adenocarcinomas of the lung. *Cancer Res* **66**, 7466-7472 (2006).

9. Bueno R*, et al.* Multi-Institutional Prospective Validation of Prognostic mRNA Signatures in Early Stage Squamous Lung Cancer (Alliance). *J Thorac Oncol* **15**, 1748-1757 (2020).
