# Supplementary material for: Single-cell analysis reveals prognostic fibroblast subpopulations linked to molecular and immunological subtypes of lung cancer
Source: Nat Commun. 2023 Jan 31;14:387. doi: 10.1038/s41467-023-35832-6 (PMC9889778; doi:10.1038/s41467-023-35832-6)
Supplement: Supplementary file 3 — Description of Additional Supplementary Files [file 41467_2023_35832_MOESM3_ESM.pdf]

Single-cell analysis reveals prognostic fibroblast subpopulations linked to molecular and immunological subtypes of lung cancer

Description of Additional Supplementary Files

File name: Supplementary Data 1
Description: Single-cell RNA-seq dataset clinicopathological information

File name: Supplementary Data 2
Description: Fibroblast subpopulation differential expression analysis results.

File name: Supplementary Data 3
Description: Fibroblast subpopulation REACTOME Pathway GSVA results.

File name: Supplementary Data 4
Description: Fibroblast subpopulation CAF gene signature GSVA results.

File name: Supplementary Data 5
Description: Intra fibroblast subpopulation control vs tumour differential expression analysis results.

File name: Supplementary Data 6
Description: Additional mxIHC images related to Figure 3b.

File name: Supplementary Data 7
Description: MxIHC dataset clinicopathological information.

File name: Supplementary Data 8
Description: Whole slide images H&E stained tissue sections for all NSCLC cases analysed by mxIHC.

File name: Supplementary Data 9
Description: Additional mxIHC images related to Figure 3c&d (LUAD)

File name: Supplementary Data 10
Description: Additional mxIHC images related to Figure 3c&d (LUSC)

File name: Supplementary Data 11
Description: Signature Matrix for CIBERSORTx mediated digital cytometry analysis.

File name: Supplementary Data 12
Description: Trajectory analysis differential expression analysis results.

File name: Supplementary Data 13
Description: Trajectory analysis Consensus module REACTOME pathway enrichment analysis.

File name: Supplementary Data 14
Description: Clinical and molecular features of bulk transcriptome datasets analysed, including digital cytometry results.

File name: Supplementary Data 15
Description: Additional mxIHC images showing solid morphology regions of LUAD tumour sections.

File name: Supplementary Data 16
Description: Gene list used for fibroblast dataset integration.
