# Supplementary material for: Single-cell analysis reveals prognostic fibroblast subpopulations linked to molecular and immunological subtypes of lung cancer
Source: Nat Commun. 2023 Jan 31;14:387. doi: 10.1038/s41467-023-35832-6 (PMC9889778; doi:10.1038/s41467-023-35832-6)

Supplementary Data 8: Whole slide images H&E stained tissue sections for all NSCLC cases analysed by mxIHC

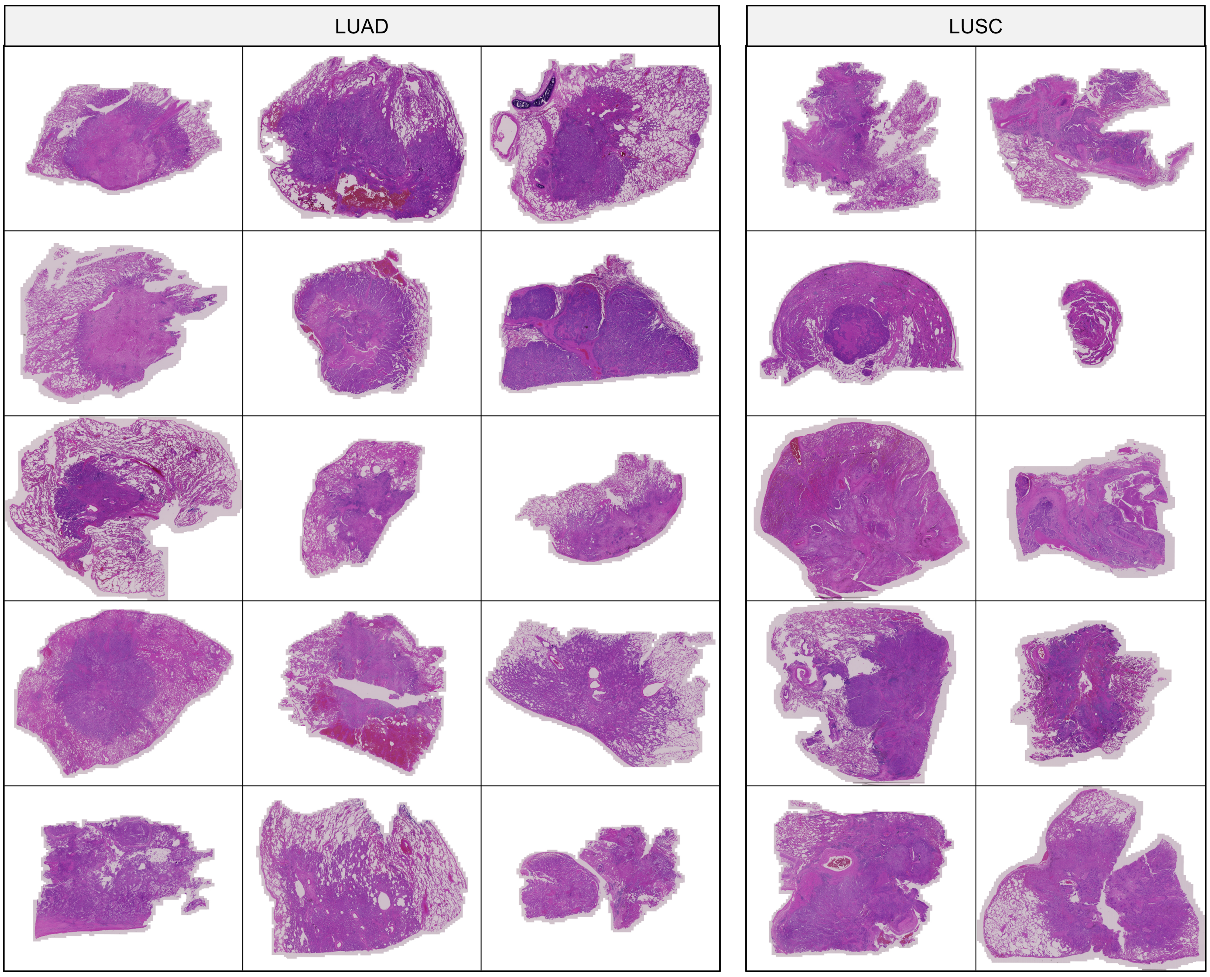

Supplement: Supplementary file 11 — Supplementary Data 8 [file 41467_2023_35832_MOESM11_ESM.pdf]
