# Supplementary material for: Single-cell analysis reveals prognostic fibroblast subpopulations linked to molecular and immunological subtypes of lung cancer
Source: Nat Commun. 2023 Jan 31;14:387. doi: 10.1038/s41467-023-35832-6 (PMC9889778; doi:10.1038/s41467-023-35832-6)
Supplement: Supplementary file 12 — Supplementary Data 9 [file 41467_2023_35832_MOESM12_ESM.pdf]

Supplementary Data 9: Additional mxIHC images related to Figure 3c (LUAD)

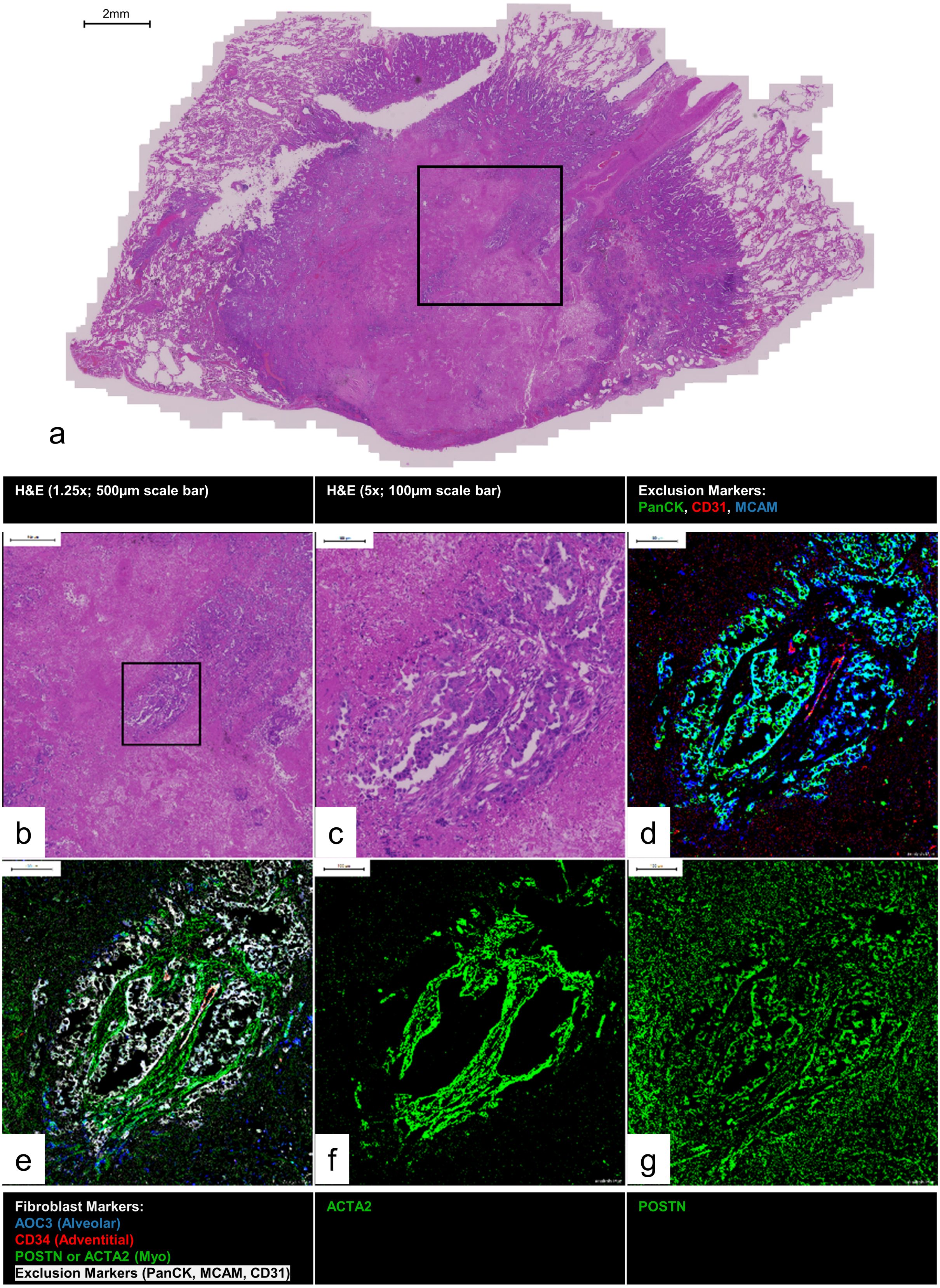

a) Whole slide image of H&E stained control lung tissue, indicating region of interest (ROI) displayed in panel b. Scale bar represents 2mm.

b) Micrograph of ROI selected from panel a, indicating ROI displayed in panels c-g.

c-g) Micrographs showing H&E and mxIHC from serial sections. Coloured as described in the associated key.

c) Shows the H&E image with no markup

d) Shows a pseudo immunofluorescence (pIF) image from mxIHC staining for exclusion markers individually coloured.

e) Shows a pseudo immunofluorescence (pIF) image from mxIHC staining for fibroblast markers (in red, green and blue) and exclusion markers (all coloured white) as indicated in the key.

f) Shows myo marker ACTA2 staining individually

g) Shows myo marker POSTN staining individually
