# Supplementary material for: Single-cell analysis reveals prognostic fibroblast subpopulations linked to molecular and immunological subtypes of lung cancer
Source: Nat Commun. 2023 Jan 31;14:387. doi: 10.1038/s41467-023-35832-6 (PMC9889778; doi:10.1038/s41467-023-35832-6)
Supplement: Supplementary file 18 — Supplementary Data 15 [file 41467_2023_35832_MOESM18_ESM.pdf]

Supplementary Data 15: Additional mxIHC images showing solid morphology regions of LUAD tumour sections (Figures provided below).

a) Whole slide image of H&E stained control lung tissue, indicating region of interest (ROI) displayed in panel b. Scale bar represents 2mm.

b) Micrograph of ROI selected from panel a, indicating ROI displayed in panels c-g.

c-g) Micrographs showing H&E and mxIHC from serial sections. Coloured as described in the associated key.

c) Shows the H&E image with no markup

d) Shows a pseudo immunofluorescence (pIF) image from mxIHC staining for exclusion markers individually coloured.

e) Shows a pseudo immunofluorescence (pIF) image from mxIHC staining for fibroblast markers (in red, green and blue) and exclusion markers (all coloured white) as indicated in the key.

f) Shows myo marker ACTA2 staining individually

g) Shows myo marker POSTN staining individually

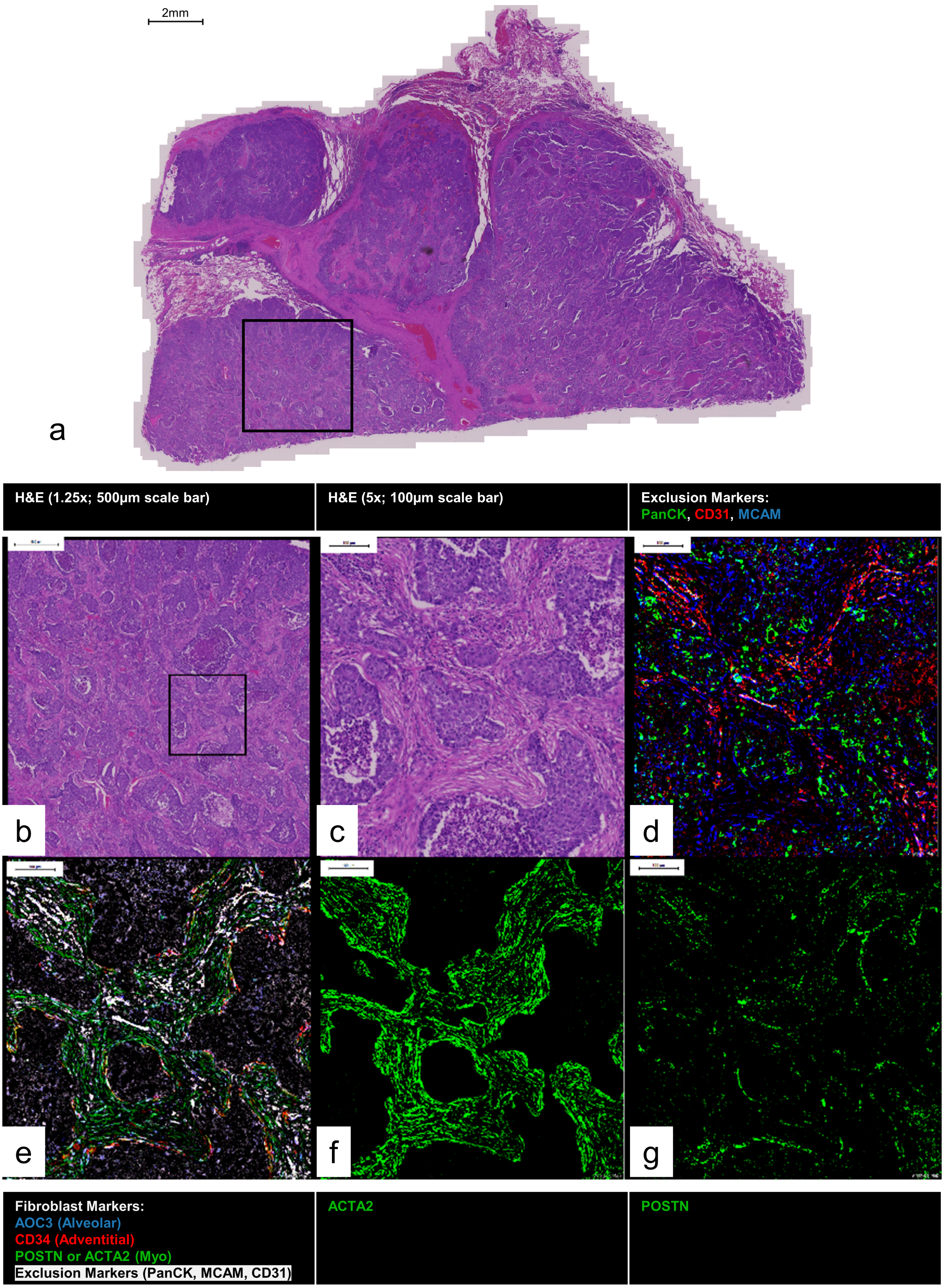
2mm
a
H&E (1.25x; 500µm scale bar)
H&E (5x; 100µm scale bar)
Exclusion Markers:
PanCK, CD31, MCAM
b
c
d
e
f
g
Fibroblast Markers:
AOC3 (Alveolar)
CD34 (Adventitial)
POSTN or ACTA2 (Myo)
Exclusion Markers (PanCK, MCAM, CD31)
ACTA2
POSTN

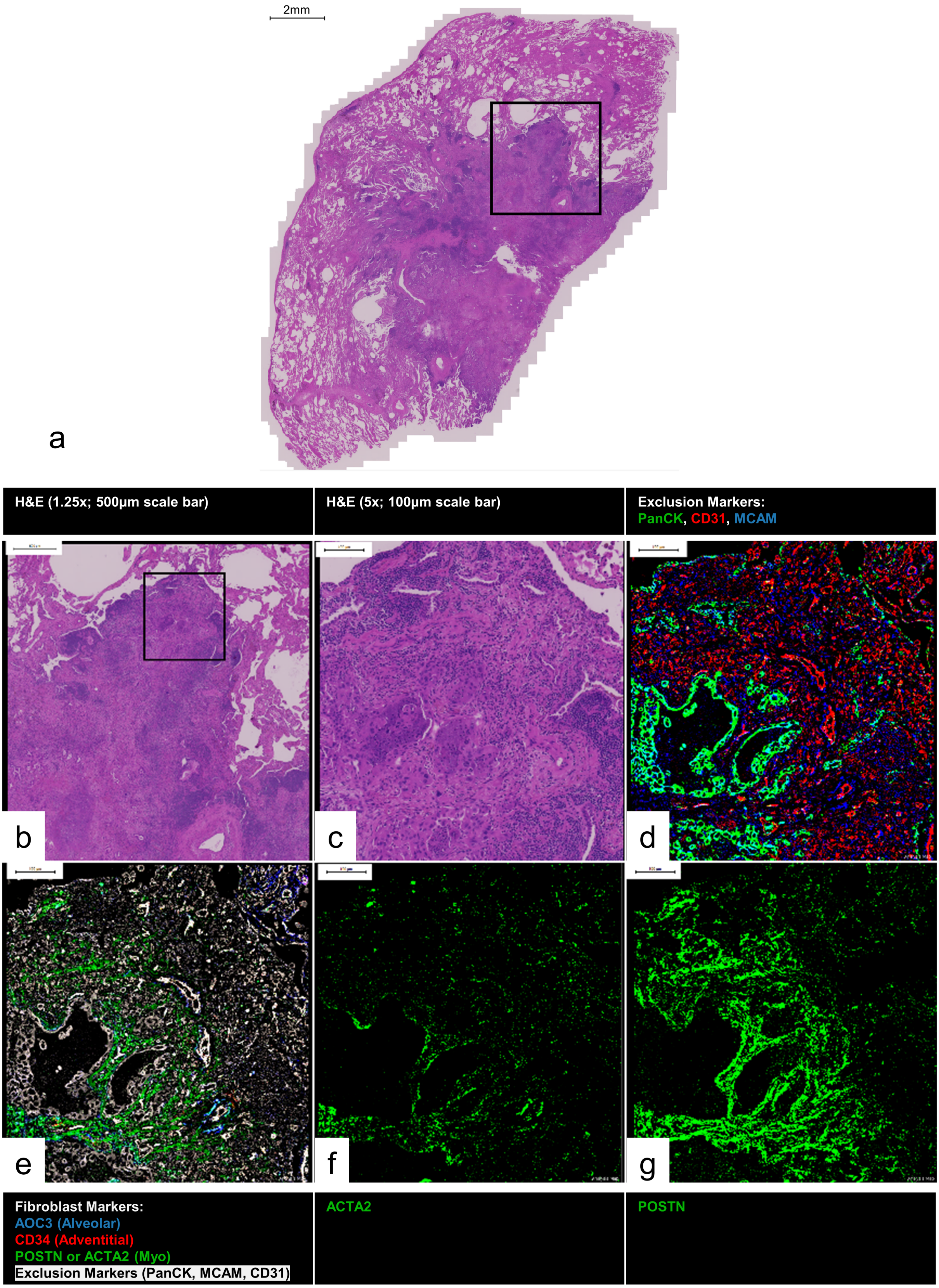
2mm
a
H&E (1.25x; 500μm scale bar)
H&E (5x; 100μm scale bar)
Exclusion Markers:
PanCK, CD31, MCAM
b
c
d
e
f
g
Fibroblast Markers:
AOC3 (Alveolar)
CD34 (Adventitial)
POSTN or ACTA2 (Myo)
Exclusion Markers (PanCK, MCAM, CD31)
ACTA2
POSTN

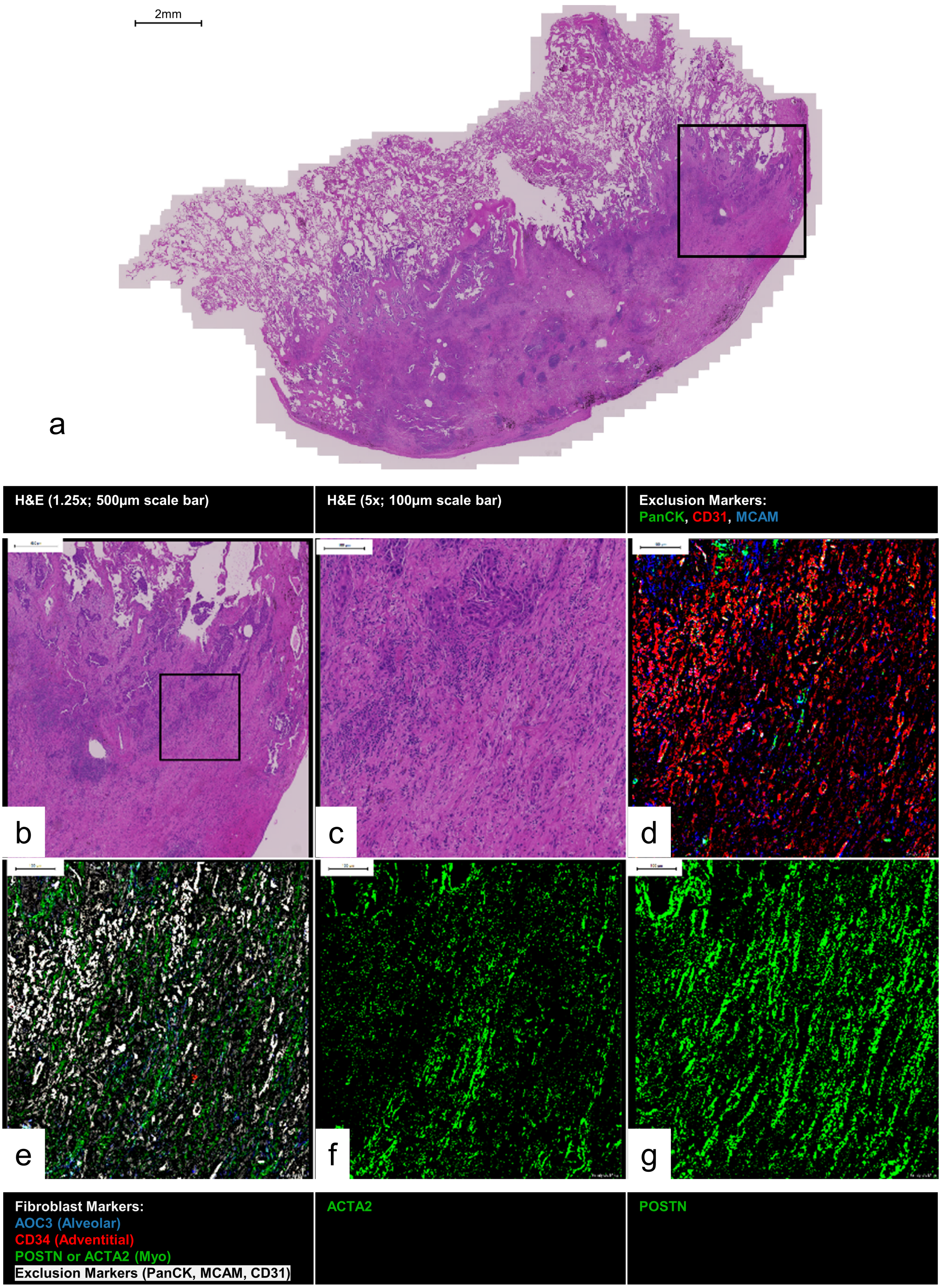
2mm
a
H&E (1.25x; 500µm scale bar)
H&E (5x; 100µm scale bar)
Exclusion Markers:
PanCK, CD31, MCAM
b
c
d
e
f
g
Fibroblast Markers:
AOC3 (Alveolar)
CD34 (Adventitial)
POSTN or ACTA2 (Myo)
Exclusion Markers (PanCK, MCAM, CD31)
ACTA2
POSTN

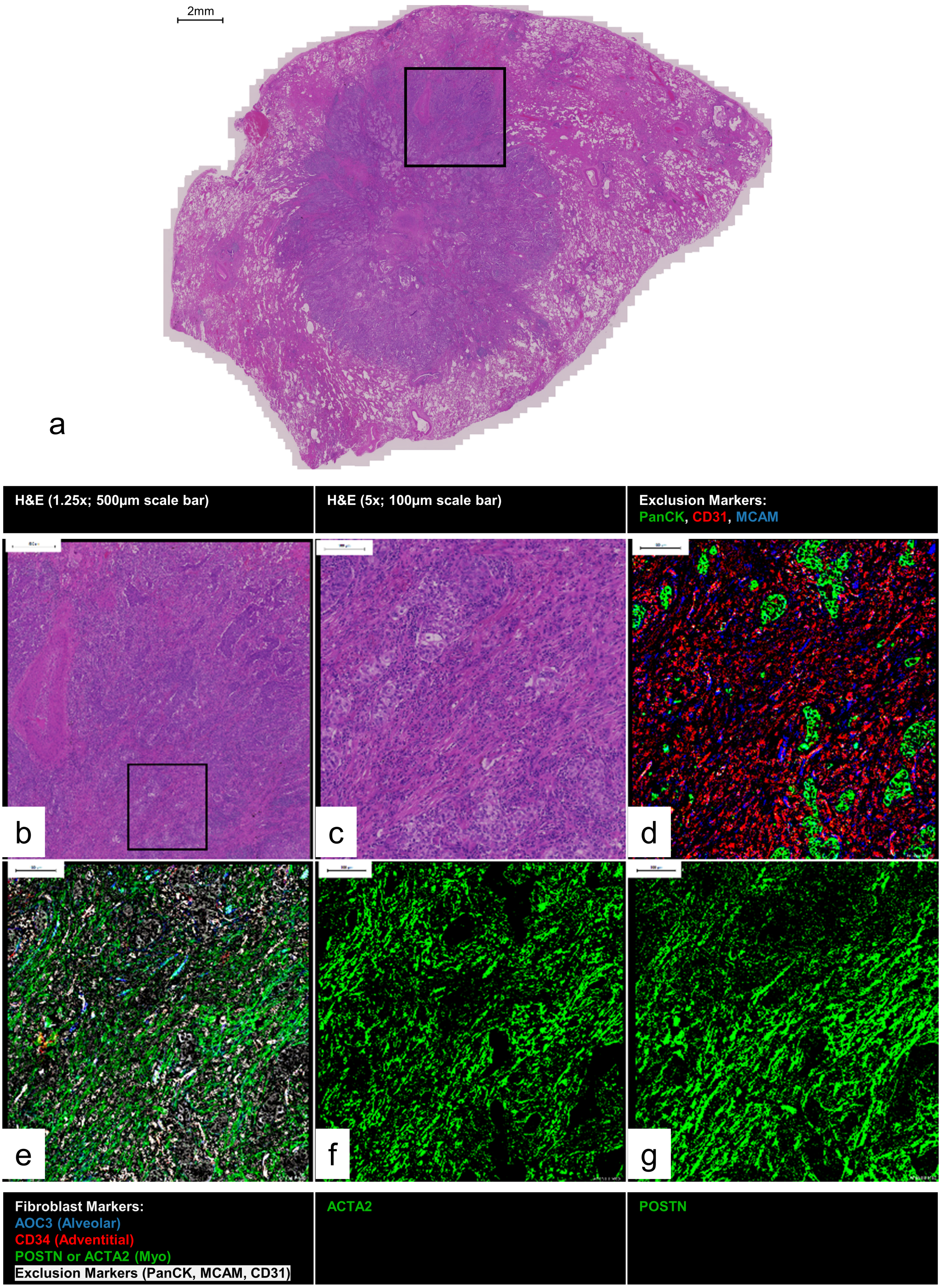

2mm
a
H&E (1.25x; 500µm scale bar)
H&E (5x; 100µm scale bar)
Exclusion Markers:
PanCK, CD31, MCAM
b
c
d
e
f
g
Fibroblast Markers:
AOC3 (Alveolar)
CD34 (Adventitial)
POSTN or ACTA2 (Myo)
Exclusion Markers (PanCK, MCAM, CD31)
ACTA2
POSTN
