# Supplementary material for: Single-cell analysis reveals prognostic fibroblast subpopulations linked to molecular and immunological subtypes of lung cancer
Source: Nat Commun. 2023 Jan 31;14:387. doi: 10.1038/s41467-023-35832-6 (PMC9889778; doi:10.1038/s41467-023-35832-6)
Supplement: Supplementary file 20 — Reporting Summary [file 41467_2023_35832_MOESM20_ESM.pdf]

nature research

Corresponding author(s): Christopher Hanley and Gareth Thomas

Last updated by author(s): Jan 4, 2023

Reporting Summary

Nature Research wishes to improve the reproducibility of the work that we publish. This form provides structure for consistency and transparency in reporting. For further information on Nature Research policies, see our Editorial Policies and the Editorial Policy Checklist.

Statistics

For all statistical analyses, confirm that the following items are present in the figure legend, table legend, main text, or Methods section.

n/a	Confirmed	
☐	☒	The exact sample size (*n*) for each experimental group/condition, given as a discrete number and unit of measurement
☐	☒	A statement on whether measurements were taken from distinct samples or whether the same sample was measured repeatedly
☐	☒	The statistical test(s) used AND whether they are one- or two-sided<br>*Only common tests should be described solely by name; describe more complex techniques in the Methods section.*
☐	☒	A description of all covariates tested
☐	☒	A description of any assumptions or corrections, such as tests of normality and adjustment for multiple comparisons
☐	☒	A full description of the statistical parameters including central tendency (e.g. means) or other basic estimates (e.g. regression coefficient) AND variation (e.g. standard deviation) or associated estimates of uncertainty (e.g. confidence intervals)
☐	☒	For null hypothesis testing, the test statistic (e.g. F, t, r) with confidence intervals, effect sizes, degrees of freedom and P value noted<br>*Give P values as exact values whenever suitable.*
☒	☐	For Bayesian analysis, information on the choice of priors and Markov chain Monte Carlo settings
☒	☐	For hierarchical and complex designs, identification of the appropriate level for tests and full reporting of outcomes
☐	☒	Estimates of effect sizes (e.g. Cohen's *d*, Pearson's *r*), indicating how they were calculated

Our web collection on statistics for biologists contains articles on many of the points above.

Software and code

Policy information about availability of computer code

Data collection	No software was used
Data analysis	The open source software environment R (v4.0.2) was utilised with the RStudio interface (v2021.09.1+372 "Ghost Orchid" Release) to perform data analysis. The packages (and versions) used are detailed below:<br>gam (v1.20); forestmodel (v0.6.2); survminer (v0.4.9); metap (v1.5); destiny (v3.2.0); Hmisc (v4.6-0); Formula (v1.2-4); survival (v3.2-11); lattice (v0.20-41); limma (v3.44.3); GSVA (v1.36.3); msigdbr (v7.4.1); ggsci (v2.9); ggpubr (v0.4.0); forcats (v0.5.1); stringr (v1.4.0); dplyr (v1.0.8); purrr (v0.3.4); readr (v2.1.2); tidyr (v1.2.0); tibble (v3.1.6); tidyverse (v1.3.1); WGCNA (v1.70-3); fastcluster (v1.2.3); dynamicTreeCut (v1.63-1); ggplot2 (v3.3.5); SeuratObject (v4.0.4); Seurat (v4.0.2); TCGAbiolinks (v2.16.4); GEOquery (v.2.56.0).<br>Multiplex immunohistochemistry image analysis was performed using Definiens Developer XD (v2.7) and Fiji (v2.3.0).<br><br>For further details the R scripts used are available on Github (https://github.com/cjh-lab/NCOMMS_NSCLC_scFibs.git).

For manuscripts utilizing custom algorithms or software that are central to the research but not yet described in published literature, software must be made available to editors and reviewers. We strongly encourage code deposition in a community repository (e.g. GitHub). See the Nature Research guidelines for submitting code & software for further information.

Data

Policy information about availability of data

All manuscripts must include a data availability statement. This statement should provide the following information, where applicable:

- Accession codes, unique identifiers, or web links for publicly available datasets
- A list of figures that have associated raw data
- A description of any restrictions on data availability

The raw scRNA-sequencing data generated in this study are available using the NCBI Gene Expression Omnibus database: GSE153935 (https://www.ncbi.nlm.nih.gov/geo/query/acc.cgi?acc=GSE153935). Additional datasets required to reproduce the analysis and figures have been published on Zenodo (https://doi.org/10.5281/zenodo.7400873). These include a Seurat object holding scRNA-sequencing data and the results of our data integration for fibroblasts isolated from multiple human lung cancer datasets (used from figure 2 onwards in our paper); a dataframe holding the histo-cytometry results from our multiplexed immunohistochemistry (mxIHC) analysis performed on whole human lung cancer tissue sections; and further ".Rdata" files required for readers to reproduce the paper's analysis and figures. Source data are also provided with this paper as indicated in the figure legends.
The publicly available datasets used in this study are available from the following sources. Bulk tissue RNA-seq (TCGA-LUAD/LUSC24, [25]) from the NIH-NCI Genomics Data commons (https://gdc.cancer.gov/access-data). The publicly available bulk tissue microarray datasets are available from the NCBI Gene expression omnibus (GEO; https://www.ncbi.nlm.nih.gov/geo/) under the following accession codes GSE72094 (https://www.ncbi.nlm.nih.gov/geo/query/acc.cgi?acc=GSE72094)[51], GSE31210 (https://www.ncbi.nlm.nih.gov/geo/query/acc.cgi?acc=GSE31210)[52], GSE68465 (https://www.ncbi.nlm.nih.gov/geo/query/acc.cgi?acc=GSE68465)53, GSE4573 (https://www.ncbi.nlm.nih.gov/geo/query/acc.cgi?acc=GSE4573)[54], GSE157009 (https://www.ncbi.nlm.nih.gov/geo/query/acc.cgi?acc=GSE157009)55, GSE157010 (https://www.ncbi.nlm.nih.gov/geo/query/acc.cgi?acc=GSE157010)[55].
The publicly available NSCLC scRNA-seq datasets are available from various online repositories: the Kim et al study data is available from NCBI GEO under the accession code GSE131907 (https://www.ncbi.nlm.nih.gov/geo/query/acc.cgi?acc=GSE131907)[16]; the Qian et al dataset is available on the ArrayExpress database at EMBL-EBI under accession codes E-MTAB-6149 (https://www.ebi.ac.uk/biostudies/arrayexpress/studies/E-MTAB-6149) and E-MTAB-6653 (https://www.ebi.ac.uk/biostudies/arrayexpress/studies/E-MTAB-6653)[32]; the Travaglini et al dataset is available on the synapse database under the accession code syn21041850 (https://doi.org/10.7303/syn21041850)[18]; the Maynard et al dataset is available as an NCBI BioProject under the accession code PRJNA591860 and processed data was accessed at the following link (https://github.com/czbiohub/scell_lung_adenocarcinoma)[34], the Bischoff et al dataset is available as a Code Ocean capsule under the following doi https://doi.org/10.24433/CO.0121060.v131. The publicly available IPF dataset used is available from NCBI GEO under the accession code GSE136831 (https://www.ncbi.nlm.nih.gov/geo/query/acc.cgi?acc=GSE136831)[15]. The publicly available PDAC dataset is available as an NCBI BioProject under the accession code PRJCA001063 (https://ngdc.cncb.ac.cn/bioproject/browse/PRJCA001063)[49]; The publicly available CRC dataset is available from NCBI GEO under the accession code GSE132465 (https://www.ncbi.nlm.nih.gov/geo/query/acc.cgi?acc=GSE132465)50; the publicly available HNSCC dataset is available from NCBI GEO under the accession code GSE103322 (https://www.ncbi.nlm.nih.gov/geo/query/acc.cgi?acc=GSE103322)[29].

Field-specific reporting

Please select the one below that is the best fit for your research. If you are not sure, read the appropriate sections before making your selection.

☒ Life sciences ☐ Behavioural & social sciences ☐ Ecological, evolutionary & environmental sciences

For a reference copy of the document with all sections, see nature.com/documents/nr-reporting-summary-flat.pdf

Life sciences study design

All studies must disclose on these points even when the disclosure is negative.

Sample size	12 surgically resected NSCLC (5 LUAD and 7 LUSC) tumour samples and 5 paired control tissue samples were analyzed at a single-cell level. 25 FFPE archived NSCLC samples (15 LUAD and 10 LUSC) were analyzed by multiplexed IHC. No statistical methods were used to predetermine sample size. Sample sizes were chosen based on qualitative estimates from previous studies.<br>For single-cell analysis approximately 1000 cells per sample were sequenced (based on cDNA concentration measurements), this sample size was chosen following optimization experiments to determine the optimal number of cells that could be sequenced at an adequate read depth using the Drop-Seq platform.
Data exclusions	Cells were only excluded from the presented analysis when they failed to meet multiple quality control criteria, which are described in detail in the methods section.<br>First, we used a random forest classifier to exclude empty droplets. We then identified outliers for the fraction of reads mapping to mitochondrial genes (> 2 median absolute deviations; MADs) to exclude apoptotic cells.<br>Low quality cells were also removed from the lymphocyte cluster using the AddModuleScore function to calculate the average expression of previously described T-cell markers (TRBC2, CD3D, CD3E, CD3G, CD2, IL7R, CD8A) and NK cell markers (FGFBP2, SPON2, KLRF1, NKG7, PRF1, KLRD1). This cluster was then filtered further to remove cells negative for both gene signatures.<br>Finally following in silico isolation of fibroblasts doublet exclusion was performed after initial clustering identified small clusters of cells marked by immune cell markers, likely to represent fibroblast/immune cell doublets or contaminating immune cells.
Replication	scRNA-seq experiments were performed on >=5 biological replicates (5 control, 5 LUAD and 7 LUSC) replicated. Multiplexed immunohistochemistry experiments were on >=10 biological replicates (15 LUAD and 10 LUSC). Due to the limited tissue available from these clinical samples no technical replication was performed. To ensure reproducibility of the findings presented in this study, we also performed a meta-analysis of scRNA-seq and bulk transcriptome datasets (utilising publicly available data from previous studies) and validated all major findings with orthogonal experimental approaches. Meta-statistics from these analyses are presented in the relevant results sections.
Randomization	Participants were not assigned experimental groups, therefore no randomization was performed.

Blinding	As above blinding was not relevant to our study as participants were not assigned experimental groups.

Reporting for specific materials, systems and methods

We require information from authors about some types of materials, experimental systems and methods used in many studies. Here, indicate whether each material, system or method listed is relevant to your study. If you are not sure if a list item applies to your research, read the appropriate section before selecting a response.

Materials & experimental systems

n/a	Involved in the study
☐	☒ Antibodies
☒	☐ Eukaryotic cell lines
☒	☐ Palaeontology and archaeology
☒	☐ Animals and other organisms
☐	☒ Human research participants
☒	☐ Clinical data
☒	☐ Dual use research of concern

Methods

n/a	Involved in the study
☒	☐ ChIP-seq
☒	☐ Flow cytometry
☒	☐ MRI-based neuroimaging

Antibodies

Antibodies used

All antibodies used are described in Table 1 of the manuscript.
Table 1: Details of antibodies used for multiplexed Immunohistochemistry. RTU = product purchased at supplier designated "Ready to use" concentration.
Target |Clone |Supplier |Product Code |Dilution |Ag Retrieval pH
CD31 |JC70A |Agilent/Dako |IR61061-2 |5x (RTU) |HIGH
Pan-CK |AE1/AE3 |Agilent/Dako |IR05361-2 |5x (RTU)| HIGH
MCAM |Polyclonal |SIGMA/Merck |HPA008848 |500x |LOW
ACTA2 (αSMA) |1A4 |Agilent/Dako |IR61161-2 |1x (RTU) |HIGH
POSTN |Polyclonal |SIGMA/Merck |HPA012306 |50x |LOW
CD34 |QBEnd 10 |Agilent/Dako |M716501-2 |50x |LOW
AOC3 |#393112 |R&D Systems |MAB3957 |500x |LOW
HSPA1A |3A3 |Santa Cruz |sc-32239 |250x |LOW

Validation

All antibodies used were commercially available and validated by the manufacturer for use as described in this study. In house staining protocol optimization was performed to national diagnostic standards (NEQAS), using manufacturer recommended positive control tissue samples assessed by a consultant pathologist. Automated immunohistochemistry was performed in a clinically-accredited pathology laboratory.
Manufacturer validation statements are provided below and further details are available at the linked web pages:
CD31 - Validated for in vitro diagnostic use (https://www.agilent.com/store/fr_FR/Prod-IR61061-2/IR61061-2)
Pan-CK - Validated for in vitro diagnostic use (https://www.agilent.com/store/productDetail.jsp?catalogId=IR05361-2)
MCAM - Enhanced Validation performed by the human protein atlas (HPA) project including: IHC tissue array of 44 normal human tissues and 20 of the most common cancer type tissues; and Protein array of 364 human recombinant protein fragments (https://www.sigmaaldrich.com/GB/en/product/sigma/hpa008848)
ACTA2 - Validated for in vitro diagnostic use (https://www.agilent.com/store/productDetail.jsp?catalogId=IR61161-2)
POSTN - Enhanced Validation performed by the HPA project including: IHC tissue array of 44 normal human tissues and 20 of the most common cancer type tissues; and Protein array of 364 human recombinant protein fragments (https://www.sigmaaldrich.com/GB/en/product/sigma/hpa012306)
CD34 - Validated for in vitro diagnostic use (https://www.agilent.com/store/ja_JP/Prod-M716501-2/M716501-2)
AOC3 - Enhanced IHC Validation performed by the HPA project using HC tissue array of 44 normal human tissues and 20 of the most common cancer type tissues (https://www.proteinatlas.org/ENSG00000131471-AOC3/antibody)
HSPA1A - Validated for research use including FFPE IHC (https://datasheets.scbt.com/sc-32239.pdf)

Human research participants

Policy information about studies involving human research participants

Population characteristics	Sc-RNA-seq: The cohort analyzed consisted of 12 NSCLC patients, population characteristics described in Supplementary Data 1.<br>MxIHC: The cohort analyzed consisted of 25 NSCLC patients, population characteristics described in Supplementary Data 7.
Recruitment	Patients undergoing surgery at Southampton General Hospital were prospectively recruited (TargetLung study). Informed consent was obtained from patients (or their legal guardians). This recruitment/analysis strategy for scRNA-seq analysis necessitates that patients were deemed clinically fit enough to undergo surgical resection (to enable fresh samples to be obtained), which may have led to the omission of patients with aggressive and late stage disease. However, patients with later stage disease were included in the publicly available data analysed in this study, therefore these recruitment criteria should not impact the results presented. Sex and/or Gender were not considered in the study design and analyses based on these characteristics were not performed due to insufficient statistical power for deriving meaningful associated conclusions.
Ethics oversight	The Southampton and South West Hampshire Research Ethics Committee approved the study, and written informed consent was obtained from all subjects (TargetLung study: REC number 14/SC/0186).

Note that full information on the approval of the study protocol must also be provided in the manuscript.
